# Quantitative analysis of nasal transcripts reveals potential biomarkers for Parkinson’s disease

**DOI:** 10.1038/s41598-019-47579-6

**Published:** 2019-07-31

**Authors:** Hyojung Kim, Seok-Jae Kang, Young Mi Jo, Min Song Kim, Yunjong Lee, Seok-Hyun Cho, Hee-Tae Kim

**Affiliations:** 1 Division of Pharmacology, Department of Molecular Cell Biology, Samsung Biomedical Research Institute, Sungkyunkwan University School of Medicine, Suwon, Republic of Korea; 2 Department of Neurology, H+ Yang-Ji Hospital, Seoul, Korea; 3 Department of Otorhinolaryngology-Head and Neck Surgery, Hanyang University College of Medicine, Seoul, Korea; 4 Department of Neurology, Hanyang University College of Medicine, Seoul, Korea

**Keywords:** Assay systems, Diagnostic markers, Parkinson's disease

## Abstract

Patients with Parkinson’s disease (PD) oftentimes develop olfactory dysfunction in their early stages, converting the nasal environment into a useful source of potential biomarkers. Here we determined the possible application of nasal fluid cells for PD biomarker identification. Thirty PD patients and 13 age-matched healthy controls were enrolled in this study. Messenger RNA levels of selected PD-related genes were monitored through real-time quantitative PCR. Target gene transcripts can be efficiently amplified from the cDNA library from human nasal fluid cell pellets. And subsequent analysis showed both a marked downregulation of parkin transcripts and an upregulation of AIMP2 in PD patients when compared to controls (cutoff value = 1.753 for with 84.2% sensitivity and 84.6% specificity; 0.359 for parkin with 76.7% sensitivity and 76.9 specificity). Moreover, alteration pattern of parkin and AIMP2 in PD was distinct from another neurodegenerative disease, multiple system atrophy. Analysis in both the early and late stages of PD cases reported that parkin levels inversely correlated with PD stages. Our results validate the practical value of easily accessible nasal fluid cells and the utility of both AIMP2 and parkin as potential biomarkers for PD diagnosis.

## Introduction

Parkinson’s disease (PD) is the second most common neurodegenerative disease worldwide^1^, although its etiology is not yet fully understood. In fact, PD patients show various non-motor as well as motor symptoms. Additionally, olfactory dysfunction is known to represent an early PD symptom, preceding the manifestation of motor symptoms by years^2,3^. According to the Braak staging, evidence showing that pathologic alterations involve the olfactory bulb in PD stage 1 exist, together with the report of several autopsies revealing atrophic changes in the olfactory bulb of PD patients^4^. These findings suggest that the nasal cavity may undergo a pathophysiologic change in PD and that alterations in gene expression or abnormal protein deposition may occur.

Indeed, both the nasal epithelium and the fluid cells from the nasal cavity were used for the screening of some early biomarkers to predict the stages and the severities of various neurodegenerative and nasal inflammatory disorders^5–7^. For instance, a recent study showed that a certain microRNA serves as a stage-specific biomarker for Alzheimer’s disease, suggesting the usefulness of nasal samples for early diagnosis of this neurodegenerative disease^7^. While the nasal epithelium biopsy is a more direct source of active molecular alterations related to disease-related protein aggregation, nasal fluid cells are also in close contact with these degenerating environments and possibly reflect the pathological changes in the olfactory mucosa. Moreover, the nasal fluid cells are easily accessible and require a simple and less invasive technique for collection when compared to nasal epithelium biopsies, which represent a remarkable advantage. Nasal fluid cells mainly include epithelial, squamous, and inflammatory cells, such as mononuclear cells and neutrophils^8,9^. Although the composition of such cell populations could be affected by the nasal inflammation process, the evaluation of transcriptome changes in nasal fluid cells in correlation with disease progression could be valuable. However, this has never been assessed in association with PD diagnosis.

The molecular pathophysiology of PD has been delineated in details especially in relation to the PD-linked genetic mutations interplaying with both the dopaminergic neurodegeneration and the α-synuclein aggregation^10^. Specifically, the PD-linked recessive gene *PARKIN*, an E3 ubiquitin ligase, plays an essential role in the survival of dopaminergic neurons, mediating both the polyubiquitination and the proteasomal degradation of toxic substrates, including the aminacyl-tRNA synthetase complex interacting multi-functional protein 2 (AIMP2) and the parkin interacting substrate (PARIS)^11–14^. In fact, AIMP2 accumulation due to dysfunctional parkin leads to poly (ADP-ribose) polymerase 1 (PARP1) overactivation and dopamine cell death^15^. Along with the parkin substrate AIMP2, PARIS builds up following parkin inactivation, which leads to mitochondrial dysfunction and dopamine cells-specific toxicity^14^. Another E3 ligase for dopamine cell survival is RNF146, which was shown to maintain dopamine neurons viability via blocking PARP1 activation through selective polyubiquitination and proteasomal degradation of overstimulated PARP1^16^. Interestingly, RNF146 is downregulated in the brain of postmortem PD patients when compared to healthy controls^16^, indicating the clinical relevance of such a protein. Another critical feature of PD is Lewy body formation, an eosinophilic intracellular perinuclear protein inclusion which is mainly composed of phosphorylated α-synuclein^1,10^. Although several kinases have been implicated in the regulation of α-synuclein aggregation, the nonreceptor tyrosine kinase c-abl was recently shown to induce the Y137 phosphorylation of α-synuclein, therefore inhibiting its clearance and facilitating its aggregation^17^. Notably, a small group clinical trial on the c-abl inhibitor Nilotinib proved the inhibitor to be effective in improving the motor performance in end-stage PD patients^18^. Taken together, these genetic networks may represent potential biomarkers which are specifically altered in both the nasal environment and in the midbrain of PD patients.

In the current research, we conducted small scale screening studies to validate the presence of potential biomarkers related to PD pathogenesis in patients’ nasal fluids. Specifically, we selected the aforementioned genes known to be associated with PD pathogenesis as possible candidates. Finally, we examined the correlation between transcripts abundance found in nasal fluid cells and disease severity, thereby confirming the usefulness of these genes as potential biomarkers for the diagnosis of PD.

## Results

### General characteristics of PD patients and control subjects

A total of 30 PD patients and 13 age-matched healthy controls (age ranges: 56~89 years old for PD and 57~89 years old for controls) were enrolled in this study (Table 1). To evaluate disease-specific regulation of potential target genes, a total of 10 multiple system atrophy (MSA, age ranges: 56~77 years old) patients were also included (Table 1). The statistical comparison of age distribution among all the groups did not show a significant difference (*p* = 0.093), with an average age of 70.7 years for PD, 67.9 years for MSA, and 64.7 years old for healthy controls, respectively. Approximately an equal number of male and female subjects within PD and control group was included in the study. Classification of PD patients was based upon both the Hoehn and Yahr (H&Y) scale and the unified PD rating scores (UPDRS)^19^, which were diagnosed and evaluated by an experienced neurology specialist. The average of the H&Y scales and the UPDRS scores in the PD patient group were 2.5 and 28.2, respectively (Table 1). Specifically, the H&Y scales ranged from 1 to 5, while the UPDRS scores ranged from 5 to 62 in PD patients.

**Table 1:** Basic demographic information and clinical motor assessment scores of PD, MSA patients and healthy controls.

	Control	PD	MSA	*P* value
N	13	30	10	
Male: female	7:6	15:15	7:3	0.544
Age (years)	64.7±8.9	70.7 ± 9.4	67.9±2.2	0.093
H&Y		2.5 ± 0.2		
UPDRS		28.2 ± 3.0		

The quantitative values are presented as the mean ± SEM. The Kruskal-Wallis test was performed to compare age means among groups. Please note that any two group comparison of age distribution failed to give significant difference.

### Validation of RT-PCR in nasal fluid cells

**Figure 1:**
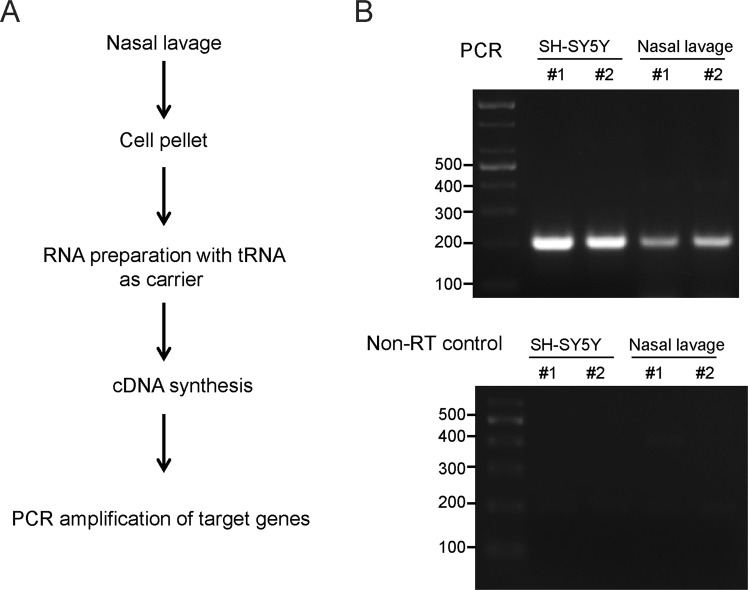
Schematic summary of both the experimental procedure and the validation of PCR amplification using the cDNA prepared from the nasal lavage cell pellet. (**A**) The detailed experimental procedure used for total RNA purification, cDNA synthesis and PCR from the human nasal lavage fluid cell pellets. (**B**) Representative gel images showing GAPDH PCR amplicon bands that were separated by agarose gel electrophoresis and visualized by red safe dye staining. The cDNA template was synthesized from the total RNA purified from both the nasal lavage cell pellets and the SH-SY5Y cells (for experimental control).

In this study, we aimed at investigating whether the transcripts from nasal fluid cell pellets obtained from each human subject were quantifiable and therefore could serve as potential biomarkers for the diagnosis of the PD status. We first created a standardized protocol for mRNA purification from nasal lavage cells and validated whether PCR assessment was applicable. Subsequently, cell pellets from each subject were obtained from the centrifugation of the nasal lavage fluid collected from a single nasal irrigation. However, variations in the cell pellet size were present among subjects. According to our preliminary assessment, approximately 197 ng (S.E. = 22.08, n = 3 large nasal fluid cell pellets) of total RNA was purified from relatively large cell pellets from the nasal lavage. To minimize RNA loss, considering the small quantity of RNA from each nasal lavage, we included a carrier yeast tRNA to maintain the consistency in the total RNA purification steps (Fig. 1A). Successively, cDNA was synthesized by reverse transcription employing the total RNA as templates and subjected to PCR using the glyceraldehyde-3-phosphate dehydrogenase (GAPDH) as a target for validation. The GAPDH amplicon by PCR was visible under UV following the separation in agarose gel, indicating successful cDNA synthesis from the total RNA prepared from the two nasal fluid cell pellets (Fig. 1B). A robust amplification of GAPDH in the cDNA was prepared from the neuroblastoma cell line (i.e., SH-SY5Y cells) as a positive control. Although the amplification of GAPDH from the nasal cDNA was weak when compared to the one from the SH-SY5Y control, it was specific. In contrast, the non-RT control failed to produce a nonspecific amplification of the potentially contaminated genomic DNA (Fig. 1B, bottom panel). Successful RT-PCR amplification was further validated for another commonly used loading control, β-actin in nasal lavage samples from control and PD patients (Supplementary Fig. S1).For these samples tested, we observed robust and consistent amplification of GAPDH which is consistent with the result in Fig. 1B (Supplementary Fig. S1). These results suggest that RT-PCR can be applied to assess target mRNA levels in nasal fluid cell pellets in humans.

### Quantitative assessment of PD-associated gene transcripts in nasal fluid cells

**Figure 2:**
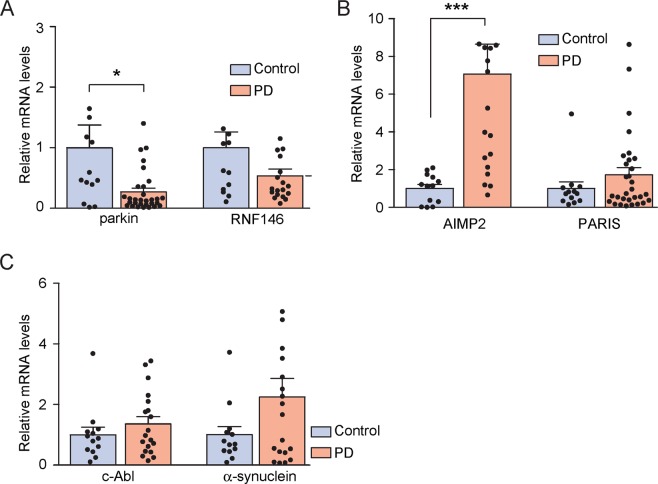
Transcript level analysis of potential biomarkers in the nasal lavage cell pellets from PD patients and healthy subjects. The relative mRNA levels of parkin/RNF146 (**A**), AIMP2/PARIS (**B**), c-Abl/ α-synuclein (**C**) in the nasal lavage cell pellets from PD patients and age-matched healthy controls determined by RTQ PCR and normalized with GAPDH levels (*n* = 13 controls, 30 PD patients for parkin and PARIS analysis; 13 controls, 19 PD patients for other genes analysis). The quantified data are expressed as the mean ± s.e.m. *P < 0.05, and ***P < 0.001, nonparametric two-tailed Mann Whitney test.

We next sought to determine the mRNA levels of PD-associated genes in nasal fluid cells from both PD patients and age-matched controls. Based upon previous PD studies^10,13–15,17^, six PD-associated genes were chosen, including neuroprotective E3 ubiquitin ligases (i.e., parkin and RNF146), parkin substrates (i.e., AIMP2 and PARIS) and Lewy pathology related genes (i.e., c-Abl kinase and α-synuclein). Each target gene was amplified through real time quantitative PCR by using the corresponding specific primers (Table 2). The abundance of each target genes was normalized by the amounts of internal loading of control GAPDH. Additionally, the relative abundance of each target gene was evaluated by the ΔΔCt method^20^, commonly used for relative comparisons of mRNA in the real-time PCR. The real-time assessment showed approximately a 73% reduction of parkin mRNA levels in PD when compared to those in age-matched controls (Fig. 2A). Interestingly, the parkin substrate AIMP2 reported a 7-fold upregulation in PD when compared to controls (Fig. 2B), although such levels are relatively maintained low with little variation in controls, as opposed to other target genes (Fig. 2). Additional genes failed to show any significant difference between the PD and control groups, although an increasing trend in both PARIS and α-synuclein was found in PD patients when compared to controls (Fig. 2A–C). To determine whether alterations of AIMP2 and parkin transcript levels are PD-specific, we further evaluated AIMP2 and parkin levels in other neurodegenerative disorder, MSA patients (Table 1). The pathological progression and affected brain circuits in MSA are different from PD but they share similar clinical motor symptoms, suggesting MSA as appropriate comparison group for PD to evaluate potential PD-specific biomarkers. Compared to age-matched healthy control, both parkin and AIMP2 mRNA levels were reduced in nasal fluid cells from MSA patients (94% reduction for parkin and 66% reduction for AIMP2, Supplementary Fig. S2). Taken together, there are distinct patterns of alterations in both AIMP2 and parkin mRNA levels in nasal fluid cells from PD, and MSA patients. Although they share parkin mRNA downregulation, only PD-derived nasal lavage samples showed upregulation of AIMP2 compared to age-matched healthy control.

**Table 2:** Sequence of the primers used for the amplification of the target gene in RTQ PCR.

Target genes	Sequence (5′ → 3′)
GAPDH	Forward: AAACCCATCACCATCTTCCAG
	Reverse: AGGGGCCATCCACAGTCTTCT
β-actin	Forward: AGAGCTACGAGCTGCCTGAC
	Reverse: AGCACTGTGTTGGCGTACAG
Parkin	Forward: CAGCAGTATGGTGCAGCGGA
	Reverse: TCAAATACGGCACTGCACTC
RNF146	Forward: ATTCCCGAGGATTTCCTTGACA
	Reverse: GCTCATCGTACTGCCACCA
AIMP2	Forward: AGGTAAAGCCCTATCACGGG
	Reverse: ACAGGTTAGACTCTTCCTGCA
PARIS	Forward: GCTGGAATTTCCGGTGTAAACC
	Reverse: GGGGTCCAAGATGGCCTCT
c-Abl	Forward: CATCACGCCAGTCAACAGTCT
	Reverse: GTACACCCTCCCTTCGTATCT
α-synuclein	Forward: AAGAGGGTGTTCTCTATGTAGGC
	Reverse: GCTCCTCCAACATTTGTCACTT

### ROC curve threshold analysis of parkin and AIMP2 mRNA levels for PD diagnosis

**Figure 3:**
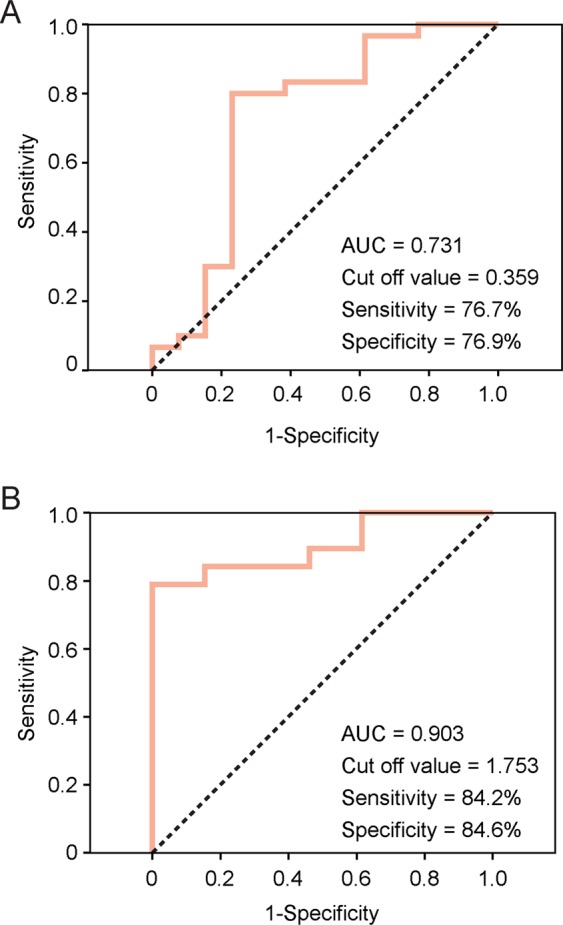
Receiver Operation Characteristic (ROC) curve analysis of sensitivity and specificity for parkin and AIMP2 as PD biomarkers. (**A**) ROC curve analysis of nasal parkin transcript levels. Cut off value, sensitivity, and specificity were noted in the inset box. (**B**) ROC curve analysis of nasal AIMP2 transcript levels. Cut off value, sensitivity, and specificity were noted in the inset box (*n* = 13 controls, 30 PD patients for parkin; 13 controls, 19 PD patients for AIMP2).

To determine the clinical application of the nasal lavage transcript analysis for PD diagnosis, a receiver operating characteristic (ROC) curve analysis was applied to parkin and AIMP2 which showed a significant alteration in PD when compared to controls. The area under curve (AUC) for parkin ROC analysis was 0.731, indicating that parkin levels represent a fair tool for PD diagnosis. The parkin cutoff value was 0.359, with the sensitivity and specificity for PD diagnosis being 76.7 and 76.9%, respectively (Fig. 3A). Additionally, subsequent analysis of AIMP2 revealed it to serve as an excellent indicator of PD diagnosis as well, with an AUC value of 0.903 (Fig. 3B). In fact, with a AIMP2 cutoff value of 1.753, PD can be diagnosed with 84.2% sensitivity and 84.6% specificity (Fig. 3B). This ROC curve analysis suggests that the quantity of AIMP2 mRNA in nasal fluid cells can be measured to sensitively diagnose PD.

### Correlational study between parkin and AIMP2 mRNA levels with UPDRS scores

**Figure 4:**
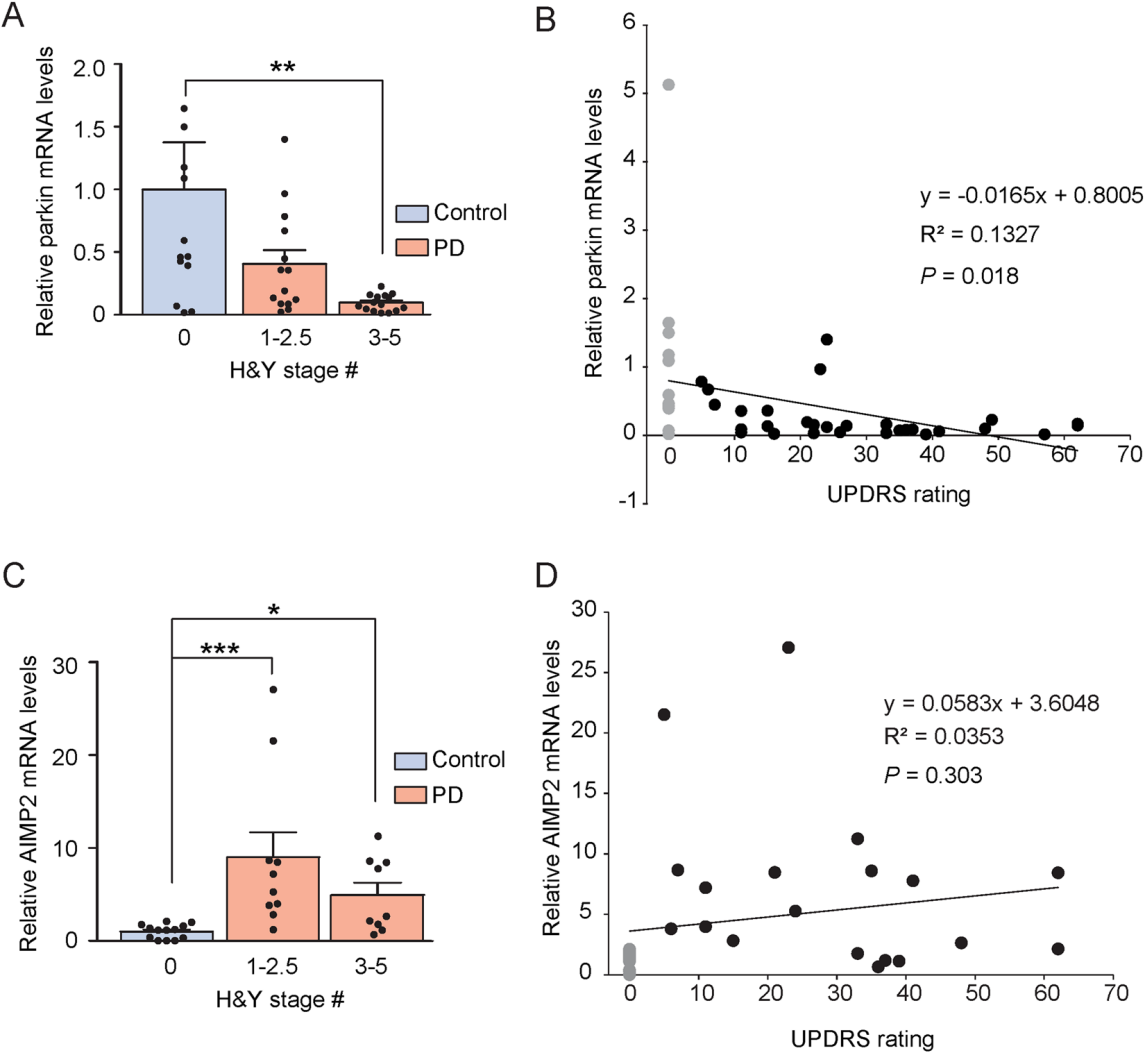
Correlation analyses between nasal AIMP2, parkin levels and clinical UPDRS scores. (**A**) The relative parkin mRNA levels in the nasal lavage cell pellets from PD patients of different H&Y stages and age-matched healthy controls determined by RTQ PCR and normalized with GAPDH levels (*n* = 13 controls, 14 PD patients with H&Y stages 1–2.5, 15 PD patients with H&Y stages 3–5). (**B**) Pearson correlation analysis between nasal parkin transcript levels and UPDRS scores (control samples in gray dot and PD samples in black dots). (**C**) Relative AIMP2 mRNA levels in the nasal lavage cell pellets from PD patients of different H&Y stages and age-matched healthy controls determined by RTQ PCR and normalized with GAPDH levels (*n* = 13 controls, 10 PD patients with H&Y stages 1–2.5, 9 PD patients with H&Y stages 3–5). (**D**) Pearson correlation analysis between nasal AIMP2 transcript levels and UPDRS scores (control samples in gray dot and PD samples in black dots). The quantified data are expressed as the mean ± s.e.m. *P < 0.05, **P < 0.01, and ***P < 0.001, nonparametric Kruskal-Wallis test followed by Dunn’s post-hoc analysis.

We next examined the correlation between parkin/AIMP2 transcript alteration and PD progression, as scored by both the H&Y and UPDRS assessment. Parkin mRNA levels gradually decreased as the PD stage advanced. The PD patient group with a H&Y scale 3~5 showed approximately a 90% reduction of parkin mRNA levels when compared to patients with a H&Y scale 0 (Fig. 4A). Moreover, we observed a significant correlation (*p* = 0.018) between relative parkin mRNA levels and UPDRS rating scores (Fig. 4B). In contrast, AIMP2 mRNA levels did not exhibit any stage-dependent progression (Fig. 4C), as they were maintained high in both the early (1~2.5 H&Y scales) and late (3~5 H&Y scales) PD stages. Specifically, the relative increase of AIMP2 mRNA was even greater in the early clinical stage of PD with approximately a 9-fold increase when compared to the 5-fold increase seen in the late stage (Fig. 4C). Furthermore, as expected, correlative changes of AIMP2 in response to different UPDRS scores were not observed among PD patients and control subjects (Fig. 4D). Similarly, we also failed to show any significant correlation between UPDRS and nasal transcript levels of RNF146, PARIS, c-Abl, and α-synuclein (Supplementary Fig. S3). Overall, these analyses revealed that parkin may be useful to confirm the PD late stage while the AIMP2 alteration is more sensitive in the early PD stage.

## Discussion

To our knowledge, the current study, which investigated some selected transcripts extracted from the nasal cavity fluid cells of PD patients and age-matched controls, was the first to report that parkin and AIMP2 mRNA levels could serve as potential biomarkers for the diagnosis of PD. Considering the emerging importance of early and sensitive diagnosis of PD, studies on biomarkers of PD are abundant, although they mainly focus on transcripts, proteins, metabolites, and amino acids in samples such as blood, serum, urine and cerebrospinal fluid (CSF). Each biological sample retains its own advantage and disadvantage. For instance, although the CSF advantage is that of being in close proximity to the brain, therefore thought to contain the most pathological information, it is relatively difficult to collect from patients. Additionally, although the blood has been studied continuously due to the relative easiness of sample collection, PD biomarkers that can be applied to clinical practice have not yet been found. In contrast, when compared to other body fluid samples, the nasal samples have some unique advantages. Firstly, nasal fluid cells collection is relatively effortless and it is simple to reduce its variation. Secondly, the olfactory bulb is one of the first organs to be affected in the early stages of PD, given that α-synuclein deposits have been identified in olfactory bulb autopsies from several PD patients in prodromal stages. Moreover, olfactory dysfunction is oftentimes observed in PD patients 5 to 10 years ahead of the manifestation of motor deficits^2,3^. In this regard, the olfactory nasal fluid cells that are parted from the nasal epithelium may reflect the pathological alterations occurring in PD patients’ olfactory bulb. Therefore, the genetic alterations of the nasal fluid cells could represent potential biomarkers for PD diagnosis. Previous studies on nasal fluid cells were limited to inflammatory disorders, including infections or allergic rhinitis^5,21^, whereas research related to neurodegenerative disease such as PD have not yet been attempted. In the current study, we first demonstrated experimentally the possibility of measuring transcripts by using real-time quantitative PCR in the nasal fluid cells obtained from PD patients and age-matched control subjects through a single nasal lavage collection. Furthermore, the usage of a carrier yeast tRNA enabled stable and reproducible collection of RNAs from single collected nasal lavage cell pellets. Moreover, the employment of real-time quantitative PCR allowed the amplification of multiple target genes and the comparison of their relative levels between the two groups. Given our straightforward and highly quantitative experimental strategy for the single collection of nasal lavage fluid samples, it possesses extensive clinical applicability and reliability.

In the present study, we measured the levels of several selected gene transcripts in the nasal fluid cells and analyzed their difference between controls and PD patients. While most of the genes failed to show a significant difference, the messenger levels of AIMP2 and parkin were differentially regulated between the two groups. It is important to note that although the AIMP2 levels are maintained at low levels in control subjects with relatively low variation, they were increased in both the early and late PD stage when compared to the healthy group. Specifically, the increase seen in the initial stage was more pronounced, while a slight decrease was observed thereafter. Given these results, we believe that AIMP2 in the nasal cavity may be a more sensitive indicator for the early diagnosis of PD. In contrast, although parkin levels are quite variable even in control subjects, a stage dependent reduction of parkin mRNA was found. Therefore, this finding suggests that parkin mRNA levels in the nasal fluid can predict disease progression, possibly indicating its applicability to reflect disease improvement with potential therapeutic strategies. It is quite striking that nasal mRNA levels of parkin and AIMP2 were also altered in MSA patients, although the pattern of alteration of AIMP2 was different between these two neurodegenerative disorders. It could be due to the presence of shared pathological and biochemical alterations in PD, and MSA to some extent. Indeed, MSA postmortem brains exhibits oligodendrocytic α-synuclein pathological aggregation in more wide brain areas^22^. PD patients also develop pathological α-synuclein pathological propagation from lower brain stem to midbrain and finally to the cortex according to Braak staging^1^. The common clinical or pathological features in these disorders may have contributed to the alteration of the genes, parkin and AIMP2. However, further investigations are needed to fully understand the molecular mechanisms underlying the differential regulation of AIMP2 during the progression of PD, and MSA. Moreover, it would be interesting to evaluate whether alterations on AIMP2 and parkin mRNA levels are movement disoder specific by comparing with other neurodegenerative cognitive disorders like Alzheimer’s disease.

Furthermore, our results reported parkin mRNA levels to be decreased in sporadic PD when compared to the healthy group. Mutations in parkin, an E3 ubiquitin ligase, are the most common cause of autosomal recessive PD, given that it has been proposed to regulate a variety of processes, including proteasomal degradation, receptor trafficking, gene expression and mitochondrial quality control^10,12^. Additionally, dysfunction of parkin has been implicated in PD pathogenesis, which has been extensively studied, with a focus on its posttranslational modifications which impair its E3 ligase functions. However, several studies regarding the transcriptional control on this neuroprotective E3 ligase have also been conducted. For example, mild endoplasmic stress was shown to stimulate the transcription of parkin through the binding of ATF4 transcription factor on the parkin promoter^23^. Moreover, parkin expression is regulated by the CREB transcription factor following glucocorticoid receptor activation^24^. Further, the parkin promoter was reported to be subject to methylation in a variable extent, although this was not found to be clinically correlated with PD^25^. Moreover, no reports or description of parkin alteration in the olfactory bulb or the nasal epithelium have been described, making it unclear the reason why such a reduction in parkin in the nasal fluid occurs during PD progression. Since parkin is an important neuroprotective E3 ligase, its downregulation in the nasal fluid cells may indicate the pathological processes of PD pathogenesis in the periphery.

Parkin substrates accumulate in pathological situations where parkin is inactivated, such as in patients with parkin mutations, sporadic PD, parkin knockout mice, and following 1-methyl-4-phenyl-1,2,3,6-tetrahydropyridine (MPTP) intoxication in mice^12,13,26^. In the present study, we monitored the mRNA levels of two parkin substrates, namely AIMP2 and PARIS that were observed an elevation in protein levels in brains affected by PD pathologies^14,27^. Among these two substrates, only the AIMP2 messenger levels were increased in the nasal fluid cells from PD patients. It is not clear whether PARIS and/or AIMP2 proteins are accumulated in the nasal fluid cells where parkin expression was reduced. The small amount of nasal fluid cell pellets hampered our analysis of protein levels detection of parkin substrates. AIMP2 is a pathological parkin substrate present in Lewy body inclusions in PD, which was reported to accumulate in the ventral midbrain of parkin knockout mice and in the postmortem brain of patients with the parkin mutation or sporadic PD^11,27^. Although previous studies on AIMP2 regulation were limited to the proteasomal degradation by parkin, our finding is the first to suggest the potential transcriptional regulation of AIMP2 in PD pathogenesis. Further studies should compare the AIMP2 transcriptional control in both the ventral midbrain and the nasal environment in PD progression.

Although our unprecedented study indicated the value of nasal fluid cells to be useful for PD diagnosis, it is widely unknown how the PD pathologies are reflected in the nasal fluid samples that are quite distant from the brain sites of major lesion in PD. Nasal fluid cells encompass epithelial cells, squamous cells and inflammatory cells, such as mononuclear cells and neutrophils^8^. It is likely that the nasal environment during PD pathogenesis following olfactory dysfunction may impact the gene regulation of these cell types. Supporting this notion, a report showing the differential gene expression of monocytes between early stage PD patients and controls is present^28^.

Since we validated the possibility of sensitively measuring the transcripts in nasal fluid cells, our detection platform could be extended to examine additional biomarkers. With regards to the translational research, PD mouse models with olfactory dysfunction could undergo transcriptome analysis of their olfactory bulb and nasal epithelium. Additionally, candidate biomarkers gained from unbiased transcriptome analysis could be easily determined for clinical nasal fluid cell samples, given its relevance, based upon our RTQ-PCR based screening. Evaluation of more disease biomarkers seems imperative to more accurate diagnosis and classification of similar movement disorders. This notion is supported by our findings that showed distinct patterns of AIMP1-parkin mRNA alteration among PD, and MSA groups. Parkin mRNA reduction was seen for both PD and MSA cases, while AIMP2 mRNA elevation was observed in only PD when compared to control. This finding suggests the value of monitoring multiple target genes in distinguishing clinically similar disorders. Finally, further large-scale cohort studies, including the projective analysis of potential PD patients over time, is needed to provide more definitive evidence that AIMP2 and parkin could be valuable biomarkers for early PD diagnosis.

## Materials and Methods

### Subjects

To evaluate the nasal expression of Parkinson-related markers, 30 patients with PD, 10 with MSA, and 13 controls were included in the current study (Table 1). All patients with PD were diagnosed according to the United Kingdom Brain Bank Diagnostic Criteria^29^. And the diagnosis of MSA was done according to established criteria^30^. All patients were investigated by movement disorder specialists, to keep the risk of misdiagnosis at a minimum. Controls visited the Department of Otolaryngology due to their deviated nasal septum (n = 8) or chronic rhinosinusitis (n = 5) and had no symptoms or history of neurodegenerative diseases. A significant difference of sex (P = 0.544) and age (P = 0.093) was not seen between the two groups. Written informed consent was obtained from all participants prior to the start of the investigation. This study was approved by the Institutional Review Board of Hanyang University Medical Center (No. 2018-01-002-002) and all experiments involving human subjects and samples in this study were performed in accordance with relevant guidelines and regulations.

### Nasal lavage sampling

Nasal lavage fluids were sampled through the suction collector following two puffs of sterile normal saline solution into each nasal cavity (total 10 μl). Subsequently, they were immediately transferred to the laboratory bench. After the removal of crust and debris with a cell strainer (100 μm, SPL, Pocheon, Gyeonggi-do, South Korea), the fluids were centrifuged at 6,000 rpm for 5 minutes. The cell pellets were resuspended in 1 ml of Trizol and stored at −80 °C until analysis.

### Total RNA extraction and PCR

Total RNA was extracted from the nasal lavage cell pellet using the QIAzol Lysis Reagent (cat# 79306, QIAGEN) with the addition of a carrier yeast tRNA (10 ug) followed by DNase I treatment to eliminate any trace of DNA contamination. cDNA was synthesized from purified nasal total RNA employing the First-strand cDNA synthesis kit (iScript cDNA synthesis kit, Biorad). Ct values of each gene were obtained from the SYBR green-based real-time PCR reaction by using the QuantStudio 6 flex Real-Time PCR System (Applied Biosystems). Relative mRNA expression levels of target genes were calculated through the ΔΔCt method^20^ using GAPDH as the internal loading control. The SYBR green PCR master mix (Cat# 4309155, Applied Biosystems) was used according to the manufacturer’s instructions. The primers utilized for real-time PCR amplification are summarized in the Table 2.

### Statistical analyses

Quantitative data are presented as the mean ± standard error of mean (SEM). Normality of the data was tested with the Shapiro-Wilk test. Statistical significance was assessed either via the nonparametric Mann-Whitney *U* test for a two-groups comparison or the nonparametric Kruskal-Wallis test with Dunn’s post-hoc analysis for comparison between more than three groups. Correlation was determined with the Pearson correlation analysis. All assessments were considered statistically significant when the *P* value was lower than 0.05.

## Supplementary information

Supplementary Information

## Data Availability

All data and resources in the manusucript are available upon reasonable request to the corresponding authors.
