# Supplementary material for: Quantitative analysis of nasal transcripts reveals potential biomarkers for Parkinson’s disease
Source: Sci Rep. 2019 Jul 31;9:11111. doi: 10.1038/s41598-019-47579-6 (PMC6668404; doi:10.1038/s41598-019-47579-6)
Supplement: Supplementary file 1 — Supplementary Information [file 41598_2019_47579_MOESM1_ESM.pdf]

Supporting Information

Quantitative analysis of nasal transcripts reveals potential biomarkers for Parkinson's disease

Hyojung Kim, Seok-Jae Kang, Young Mi Jo, Min Song Kim, Yunjong Lee, Seok-Hyun Cho, Hee-Tae Kim

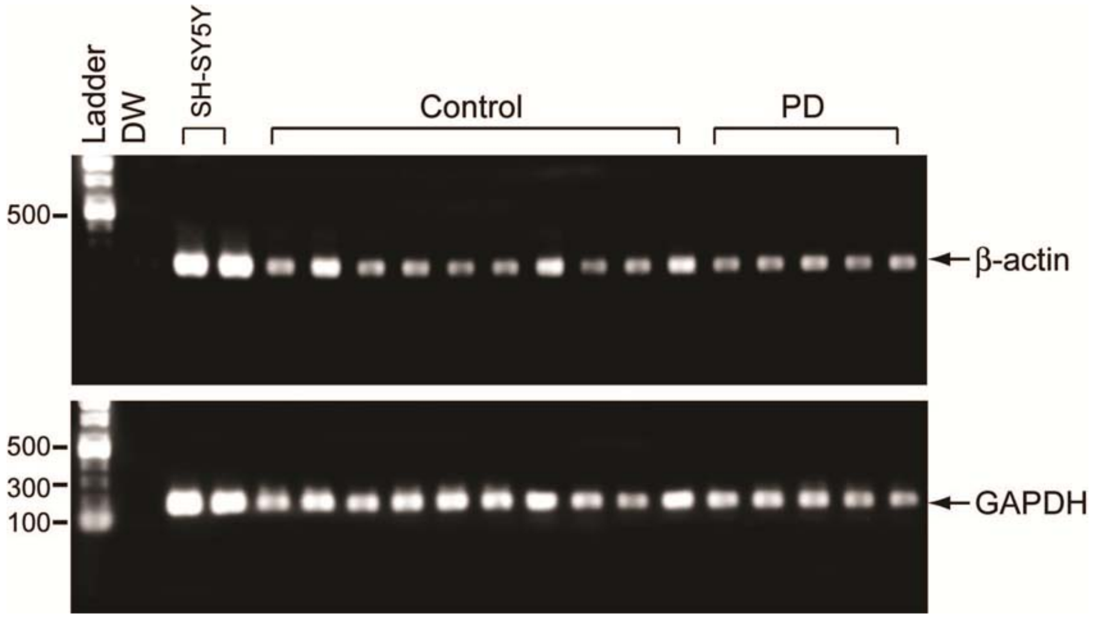

Supplementary Figure S1. Validation of PCR amplification of GAPDH and β-actin using the cDNA prepared from the nasal lavage cell pellet.

Gel images showing β-actin and GAPDH PCR amplicon bands (upper and bottom panel, respectively) that were separated by agarose gel electrophoresis and visualized by red safe dye staining. The cDNA template was synthesized from the total RNA purified from the nasal lavage cell pellets obtained from PD patients and age matched control (n = 10 control, 5 PD). DW and cDNA from SH-SY5Y cells were included as negative and positive control for PCR, respectively.

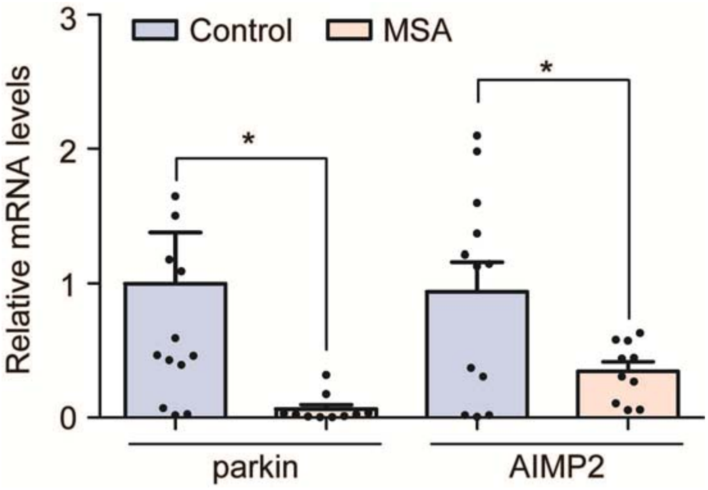

Supplementary Figure S2. Transcript level analysis of AIMP2 and parkin in the nasal lavage cell pellets from MSA patients and age-matched healthy subjects. The relative mRNA levels of parkin and AIMP2 in the nasal lavage cell pellets from MSA patients as compared to age-matched healthy controls determined by RTQ PCR and normalized with GAPDH levels (n = 13 controls, and 10 MSA patients). The quantified data are expressed as the mean ± s.e.m. $^*P < 0.05$, nonparametric two-tailed Mann Whitney test. Note that the same control group in the main Figure 2 was used to compare with MSA.

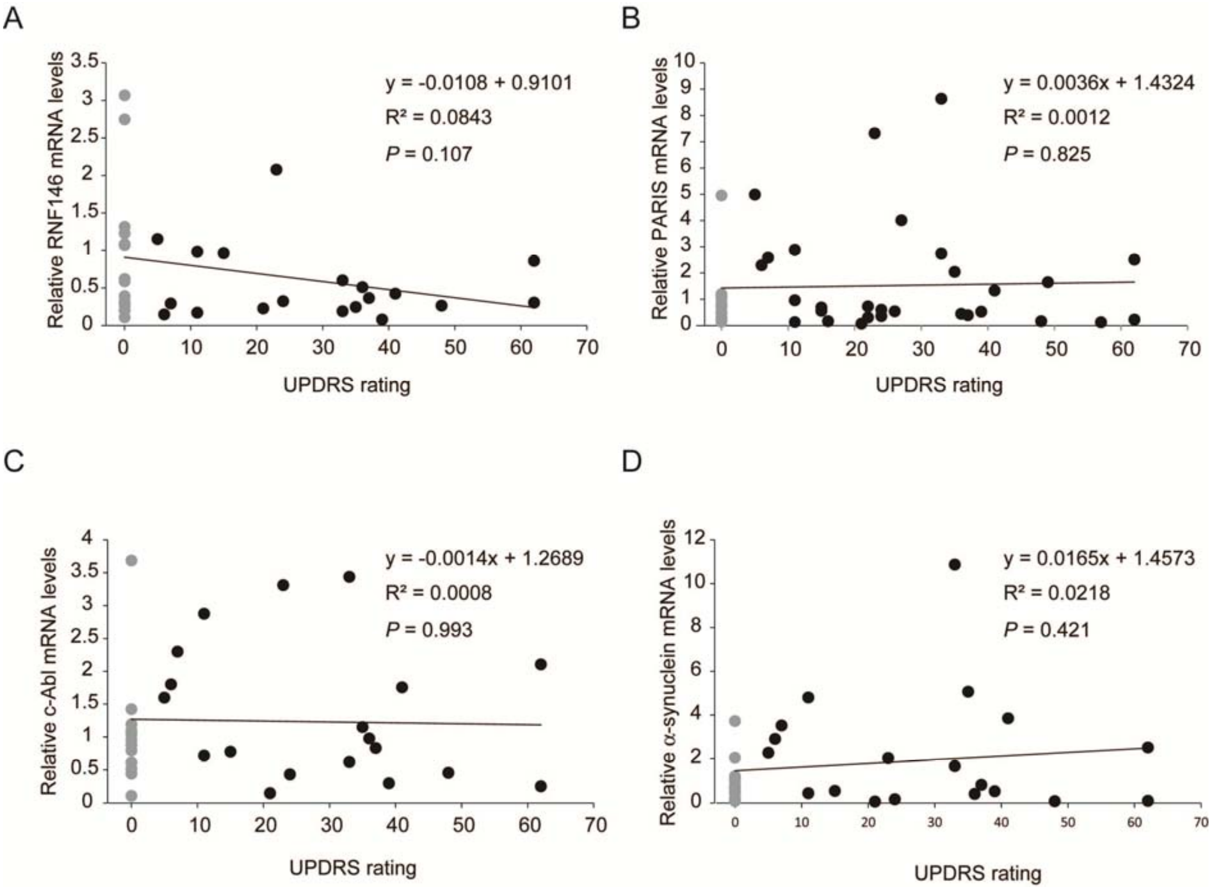

Supplementary Figure S3. Correlation analyses between nasal target transcripts and clinical UPDRS scores. The correlation between the levels of RNF146 (**A**), PARIS (**B**) c-Abl (**C**), α-synuclein (**D**) and UPDRS scores was determined by linear regression with Pearson's correlation analysis. The normalized levels of target transcripts and corresponding UPDRS score were plotted for age-matched healthy control (grey dot) and PD patients (black dot).
